# Dietary and Physical Activity Interventions for Colorectal Cancer Survivors: A Randomized Controlled Trial

**DOI:** 10.1038/s41598-018-24042-6

**Published:** 2018-04-10

**Authors:** C. F. Lee, Judy W. C. Ho, Daniel Y. T. Fong, Duncan J. Macfarlane, Ester Cerin, Antoinette M. Lee, Sharron Leung, Wynnie Y. Y. Chan, Ivy P. F. Leung, Sharon H. S. Lam, Natural Chu, Aliki J. Taylor, Kar-keung Cheng

**Affiliations:** 1 School of Public Health, The University of Hong Kong, Pok Fu Lam, Hong Kong; 2 Department of Surgery, The University of Hong Kong, Pok Fu Lam, Hong Kong; 3 School of Nursing, The University of Hong Kong, Pok Fu Lam, Hong Kong; 4 Institute for Health & Ageing, Australia Catholic University, Sydney, Australia; 5 Department of Psychology, The University of Hong Kong, Pok Fu Lam, Hong Kong; 6 School of Nursing, Hong Kong Baptist Hospital, Beacon Hill, Hong Kong; 7 School of Professional and Continuing Education, The University of Hong Kong, Pok Fu Lam, Hong Kong; 8 Department of Dietetics, Queen Elizabeth Hospital, Yau Ma Tei, Hong Kong; 9 Institute of Applied Health Research, University of Birmingham, Birmingham, United Kingdom

## Abstract

There has been evidence on the protective effects of diets high in fiber and low in red and processed meat (RPM), and physical activity (PA) against colorectal cancer (CRC) development, but that against CRC recurrence has been limited. This study evaluated the efficacy of a behavioral program comprising dietary and PA interventions in improving Chinese CRC survivors’ lifestyle. A 2 × 2 factorial randomized controlled trial of 223 CRC patients (82 females, mean age 65), randomly assigned to receive dietary, PA or both interventions, or usual care for 12 months, and assessed every 6 months for 24 months. Primary outcomes included two dietary and two PA targets. Secondary outcomes included changes in dietary consumptions and PA levels. Dietary interventions significantly increased the odds of achieving the targets of consuming less RPM at all time-points (OR 3.22–4.57, all p < 0.01) and refined grain (RG) at months 6 (OR 3.13, p = 0.002) and 24 (OR 2.19, p = 0.039), and reduced RPM (2.49–3.48 servings/week, all p < 0.01) and RG (0.31–0.5 servings/day, all p < 0.01) consumptions. Patients receiving PA interventions potentially spent more time on moderate-to-vigorous PA. This study demonstrated the efficacy of a behavioral program in improving dietary habits of Chinese CRC survivors.

## Introduction

Colorectal cancer (CRC) was the third most common cancer type and the fourth largest cause of cancer mortality in the world, with 1.4 million new cases and almost 700,000 deaths in 2012^1^. In Hong Kong, CRC was ranked first and second by incidence and mortality, respectively, in 2014^2^. These figures called for effective interventions that would prevent CRC and improve cancer outcomes in survivors.

The World Cancer Research Fund summarized evidence from observational studies and concluded that low dietary fiber, and high red and processed meat (RPM) intakes were associated with higher risk of CRC, whilst physical activity (PA) protected against developing colon cancer^3^. Importantly, dietary control and PA are non-pharmacological and non-invasive interventions that appeal to CRC patients who have had invasive cancer treatments. However, only a few randomized controlled trials (RCTs) have evaluated the efficacy of behavioral interventions in modifying the dietary and PA habits of CRC survivors. In 2011, a meta-analysis of RCTs on PA for cancer survivors identified only 3, out of 34 studies, that evaluated PA in solely CRC or colon cancer survivors^4^. Two RCTs of both PA and dietary interventions for CRC survivors have been subsequently conducted in the United States and Australia^5,6^. Also, a pilot RCT comparing two home-based PA interventions in Korean CRC survivors has been reported^7^.

All these RCTs assessed only short-term effects at 12 weeks to 12 months post-intervention. In addition, with the exception of one^7^, they were primarily conducted in Caucasian populations^5,6,8–10^ and none targeted a Chinese population. Lifestyle can vary drastically across countries due to differences in culture, infrastructure and economic situations. When compared with the West, Chinese societies have their unique culture with distinct lifestyle habits. For instance, Chinese traditionally share the main dishes in a meal with others rather than having their own portions. Hence, modifying dietary habits in Chinese would likely be more complex. Differences can also be found in PA, as in Chinese societies public transport (involving a certain amount of walking to/from transit points) is the main modality of transport^11^, whilst private cars are more common in North America^12^. These lifestyle differences necessitate the need for evaluating behavioral interventions specifically in a Chinese population.

Before assessing the efficacy of behavioral interventions on cancer outcomes, we have to assess whether the interventions may effectively modify the targeted behaviors. Therefore, we aimed to evaluate whether the dietary and PA interventions of the “Moving Bright, Eating Smart” program are effective in reducing the consumption of RPM and refined grain (RG), and increasing the PA levels in adult Chinese CRC survivors.

## Results

### Baseline characteristics

**Figure 1:**
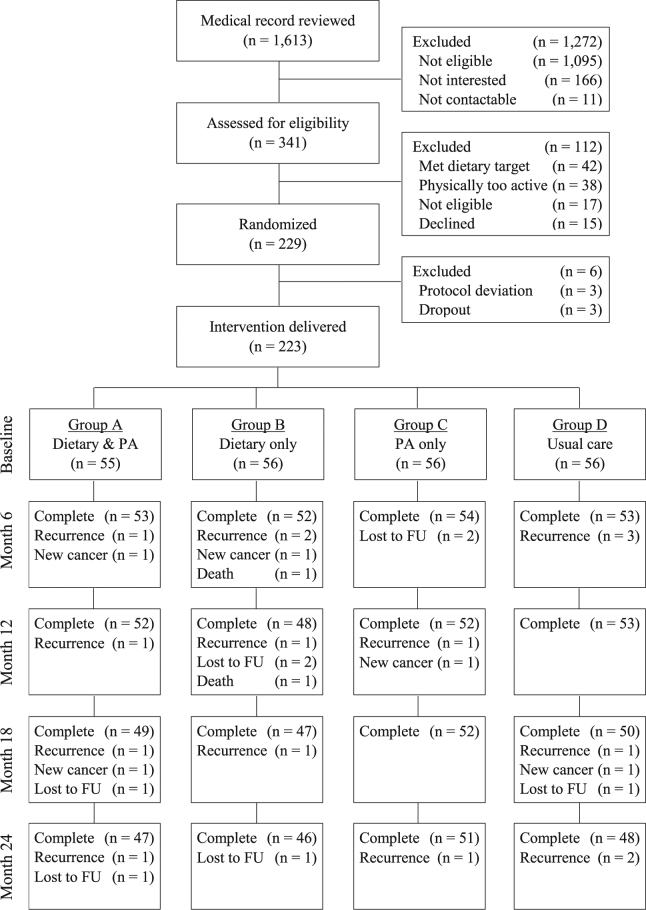
The CONSORT flowchart of the 223 colorectal cancer survivors participating in this trial.

Between 1^st^ May 2013 and 30^th^ April 2014, 229 eligible patients consented to participate in the study (Fig. 1). After randomization, 3 patients dropped out before commencing any intervention and another 3 patients were excluded due to active psychiatric illness, not residing in Hong Kong, and hereditary CRC syndrome. As a result, 223 patients received the assigned interventions. At 24 months, 192 patients completed the interventions and follow-up. Thirty-one patients dropped out from the trial due to cancer recurrence (n = 18, Group A: 4, Group B: 6, Group C: 2, Group D: 6), loss to follow-up (n = 8, Group A: 2, Group B: 3, Group C: 2, Group D: 1) and development of new cancer (n = 5, Group A: 2, Group B: 1, Group C: 1, Group D: 1). Two patients with CRC recurrence died from the disease. There were only 4 patients at stage IV and thus they were combined with the other 80 patients at stage III. All patients had surgery with 129 (57.8%) received adjuvant chemotherapy and 43 (19.3%) received adjuvant/neoadjuvant radiotherapy.

These 223 patients had a mean age of 65.2 years (standard deviation = 10.1; range = 25 to 86), and 82 (36.8%) of them were females. There were 133 (59.6%) patients with colon cancer, 89 (39.9%) with rectal cancer, and one (0.4%) with synchronous colon and rectal cancers. No significant differences in baseline characteristics among the four groups were observed (Table 1). Patients in Groups A, B and C attended an average of 94.6% of all sessions of motivational interviews, answered 72.4% of the motivational phone calls, and joined 43.5% of the group meetings.

**Table 1:** Patient characteristics at baseline.

Characteristic	Group A Dietary & PA (n = 55)		Group B Dietary only (n = 56)		Group C PA only (n = 56)		Group D Usual care (n = 56)		P
	No.	%	No.	%	No.	%	No.	%	
Age, years (mean, SD)	63.2	11.4	65.9	9.8	66.6	9.5	64.9	9.4	0.324
Sex									0.221
Male	37	67.3	34	60.7	40	71.4	30	53.6	
Female	18	32.7	22	39.3	16	28.6	26	46.4	
Education level									0.238
High school or below	7	12.7	6	10.7	11	19.6	4	7.1	
College or above	48	87.3	50	89.3	45	80.4	52	92.9	
Care giver									0.875
Self	35	63.6	35	62.5	39	69.6	39	69.6	
Spouse	12	21.8	15	26.8	13	23.2	11	19.6	
Others	8	14.5	6	10.7	4	7.1	6	10.7	
Monthly household income^†^									0.422
≤HK$10,000	21	38.2	18	32.1	20	35.7	15	26.8	
HK$10,000-HK$25,000	12	21.8	11	19.6	12	21.4	22	39.3	
>HK$25,000	19	34.5	23	41.1	19	33.9	17	30.4	
Cancer site									0.589
Colon	35	63.6	34	60.7	29	51.8	35	62.5	
Rectum	20	36.4	22	39.3	26	46.4	21	37.5	
Colon and rectum	0	0.0	0	0.0	1	1.8	0	0.0	
AJCC tumor stage									0.407
Stage I	14	25.9	9	16.1	8	14.5	12	21.4	
Stage II	24	44.4	20	35.7	27	49.1	24	42.9	
Stage III or IV	16	29.6	27	48.2	20	36.4	20	35.7	
Cigarette smoking status									0.684
Never-smoker	39	70.9	41	73.2	37	66.1	42	75.0	
Ex-smoker	14	25.5	10	17.9	16	28.6	10	17.9	
Current smoker	2	3.6	5	8.9	3	5.4	4	7.1	
Alcohol drinking habit									0.818
Never drinker	31	56.4	30	53.6	32	57.1	38	67.9	
Ex-drinker	17	30.9	17	30.4	16	28.6	12	21.4	
Current drinker	7	12.7	9	16.1	8	14.3	6	10.7	
Hypertension	18	32.7	28	50.0	20	35.7	22	39.3	0.264
Ischemic heart disease	1	1.8	5	8.9	6	10.7	2	3.6	0.165
Diabetes mellitus	8	14.5	13	23.2	6	10.7	9	16.1	0.335
Chronic respiratory diseases	3	5.5	2	3.6	0	0.0	3	5.4	0.370
Chronic renal disease	2	3.6	0	0.0	1	1.8	1	1.8	0.555
Cerebrovascular accident	2	3.6	2	3.6	0	0.0	1	1.8	0.517
Stoma status									0.922
No stoma	47	85.8	48	85.7	48	85.7	50	89.3	
Permanent or temporary sotma	8	14.5	8	14.3	8	14.3	6	10.7	
Body weight, kg (mean, SD)	62.9	12.0	62.1	11.0	61.8	10.4	61.3	10.7	0.893
BMI, kg/m^2^ (mean, SD)	23.8	3.3	24.0	3.2	23.8	3.1	23.9	3.6	0.987
Caloric intake, Kcal (mean, SD)	1530	400	1548	429	1518	367	1520	477	0.979
Dietary & PA targets									
RPM intake, servings/week (mean, SD)	9.2	6.5	8.3	5.0	7.7	4.3	8.8	7.1	0.547
RG intake, servings/day (mean, SD)	3.0	1.1	2.7	0.9	2.8	1.1	2.7	0.8	0.438
Accumulated MVPA, minutes/week (mean, SD)	534.2	329.3	498.1	316.8	460.8	239.6	473.3	267.3	0.597
Meeting red/processed meat target	14	25.5	16	28.6	10	17.9	16	28.6	0.516
Meeting refined grain target	6	10.9	7	12.5	7	12.5	5	8.9	0.922
Meeting PA general health target	47	85.5	46	82.1	46	82.1	50	89.3	0.980
Meeting PA cancer outcome target	41	74.5	34	60.7	37	66.1	42	75.0	0.614

Abbreviations: PA, physical activity; SD, standard deviation; AJCC, American Joint Committee on Cancer.

^†^Exchange rate: US$1 = HK$7.8.

### Achieving behavioral targets

Between 52.3% and 60.4% of the patients receiving the dietary interventions (Group A + B) met the RPM target (Table 2), compared to 25.9% to 31.3% of those not receiving the dietary interventions (Group C + D). There was no significant interaction effect among the dietary interventions, PA interventions and time. The overall effect of the dietary interventions on the RPM target was significant (p < 0.001), with an odds ratio (OR) of 3.88 (95% CI = 2.32–6.50). The effects of the dietary intervention were also significant at all time-points, with ORs between 3.22 and 4.57. For the RG target, the overall OR was 1.90 (1.18–3.07, p = 0.008), and significant intervention effects were also observed at months 6 (OR = 3.13 [1.51–6.48], p = 0.002) and 24 (OR = 2.19 [1.04–4.63], p = 0.039). When comparing Group A + C with Group B + D, PA interventions showed significant positive effects on the general health target overall (OR = 1.94 [1.10–3.40], p = 0.022) and at month 12 (OR = 2.45 [1.10–5.43], p = 0.028), as well as significant positive effects on the cancer outcome target overall (OR = 1.95 [1.13–3.35], p = 0.016) and at month 18 (OR = 2.38 [1.08–5.23], p = 0.018). When missing values were imputed using multiple imputation, the dietary-intervention effects remained the same, but the PA-intervention effects became insignificant. The numbers meeting the targets in each group are presented in Supplementary Table 1.

**Table 2:** Effects of dietary and physical activity interventions on achieving the behavioral targets at various time points.

Outcomes^†^	% of patients meeting the target		OR	(95% CI)	P
Dietary targets	Groups A + B	Groups C + D	Dietary interventions		
**Red/processed meat target**					
Baseline	27.0	23.2	1.19	(0.55 to 2.58)	0.661
Month 6	53.2	25.9	4.24	(1.96 to 9.17)	<0.001
Month 12	55.0	29.5	3.73	(1.74 to 7.97)	<0.001
Month 18	60.4	31.3	4.57	(2.14 to 9.76)	<0.001
Month 24	52.3	29.5	3.22	(1.50 to 6.89)	0.003
Overall^‡^	49.5	27.9	3.88	(2.32 to 6.50)	<0.001
**Refined grain target**					
Baseline	11.7	10.7	1.11	(0.41 to 3.02)	0.837
Month 6	51.4	30.4	3.13	(1.51 to 6.48)	0.002
Month 12	43.2	33.9	1.62	(0.79 to 3.35)	0.188
Month 18	39.6	35.7	1.20	(0.58 to 2.47)	0.623
Month 24	40.5	26.8	2.19	(1.04 to 4.63)	0.039
Overall^‡^	37.3	27.5	1.90	(1.18 to 3.07)	0.008
**PA targets**	**Groups A** + **C**	**Groups B** + **D**	**PA interventions**		
**PA general health target**					
Baseline	83.8	85.7	0.81	(0.31 to 2.09)	0.658
Month 6	73.0	64.3	1.81	(0.78 to 4.17)	0.166
Month 12	62.2	47.3	2.45	(1.10 to 5.43)	0.028
Month 18	67.6	56.3	2.04	(0.91 to 4.59)	0.085
Month 24	64.0	57.1	1.53	(0.68 to 3.44)	0.299
Overall^‡^	70.1	62.1	1.94	(1.10 to 3.40)	0.022
**PA cancer outcome target**					
Baseline	70.3	67.9	1.14	(0.52 to 2.50)	0.739
Month 6	68.5	57.1	1.90	(0.85 to 4.24)	0.116
Month 12	55.0	43.8	1.77	(0.81 to 3.86)	0.151
Month 18	64.0	48.2	2.38	(1.08 to 5.23)	0.031
Month 24	60.4	49.1	1.83	(0.83 to 4.02)	0.134
Overall^‡^	63.6	53.2	1.95	(1.13 to 3.35)	0.016

Abbreviations: PA, physical activity; OR, odds ratio; CI, confidence intervals.

^†^Red/processed meat target: <5 servings of red and processed meat per week, including <2 servings of processed meat.

Refined grain target: <2 servings of refined grain per day.

PA general health target ≥30 minutes of moderate-to-vigorous intensity physical activity (MVPA) 5 days a week.

PA cancer outcome target ≥60 minutes of MVPA 5 days a week.

^‡^The “overall” comparison was a test for the effect of the interventions at all follow-up time points (four levels at 6, 12, 18 and 24 months) using repeated measures mixed effects models, where the time point were regarded as a categorical variable.

### Magnitude change in dietary intake and PA level

All four groups had reduced intakes of RPM and RG and improvement in PA level at all time-points from baseline (Supplementary Table 2). The only exception was an increase in RPM consumption in Group C at month 18. Patients who underwent the dietary interventions showed significantly larger reductions in RPM intakes by 2.49 to 3.48 servings per week (p < 0.001) and RG intakes by 0.31 to 0.50 servings per day (p ≤ 0.005) when compared with those who did not receive the interventions (Table 3). The PA interventions were not significantly associated with any change in PA levels, although patients receiving the PA interventions spent 3.3 to 73.1 more minutes/week on MVPA at all time-points.

**Table 3:** Effects of dietary and physical activity interventions on changes of dietary consumption and physical activity level.

Outcomes	Mean (SD)		Difference in change from baseline	(95% CI)	P
Dietary targets	Groups A + B	Groups C + D	Dietary interventions		
**Red and processed meat intake (servings/week)**					
Baseline	8.7 (5.8)	8.2 (5.9)			
Month 6	4.3 (4.0)	7.3 (5.8)	−2.81	(−4.02 to −1.61)	<0.001
Month 12	3.8 (3.8)	7.0 (4.7)	−3.48	(−4.65 to −2.31)	<0.001
Month 18	4.5 (4.4)	7.7 (5.7)	−2.49	(−3.68 to −1.30)	<0.001
Month 24	4.6 (3.6)	7.0 (4.5)	−2.94	(−3.90 to −1.97)	<0.001
**Refined grain intake (servings/day)**					
Baseline	2.8 (1.0)	2.7 (0.9)			
Month 6	1.9 (0.8)	2.5 (1.1)	−0.50	(−0.72 to −0.27)	<0.001
Month 12	2.0 (0.9)	2.4 (1.0)	−0.31	(−0.52 to −0.09)	0.005
Month 18	2.1 (0.8)	2.3 (0.9)	−0.38	(−0.60 to −0.17)	<0.001
Month 24	2.0 (0.7)	2.4 (0.8)	−0.45	(−0.62 to −0.29)	<0.001
**PA targets**	**Groups A** **+** **C**	**Groups B** **+** **D**	**PA interventions**		
**PA level (accumulated minutes/week of MVPA)**					
Baseline	498.2 (289.8)	485.1 (290.7)			
Month 6	660.6 (317.5)	612.4 (325.8)	21.6	(−61.1 to 104.3)	0.607
Month 12	594.7 (238.3)	578.4 (291.4)	10.1	(−78.2 to 98.3)	0.823
Month 18	681.8 (309.7)	643.8 (387.6)	3.3	(−81.7 to 88.3)	0.939
Month 24	705.0 (324.0)	613.3 (321.4)	73.1	(−12.5 to 158.8)	0.094

Abbreviations: SD, standard deviation; PA, physical activity; CI, confidence intervals; MVPA, moderate-to-vigorous intensity physical activity.

In the subgroup of 49 patients who had <300 minutes of MVPA per week at baseline, PA interventions did not significantly improve the two PA targets (Table 4). However, patients who received the PA interventions had significantly larger increases in PA at months 6 (difference = 174.2, [34.7–313.7], p = 0.015) and 18 (179.0 [36.6–321.3], p = 0.014) than those who did not receive the PA interventions.

**Table 4:** Subgroup analysis of patients who did not meet the target of physical activity cancer outcome at baseline.

Outcomes^†^	PA interventions		PA-intervention effects		
	Received (n = 22)	Not received (n = 27)			
	No. (%) of patients meeting the target		Odds ratio	(95% CI)	P
**PA general health target**					
Month 6	15 (68.2)	17 (63.0)	1.67	(0.31 to 9.09)	0.551
Month 12	12 (54.5)	11 (40.7)	4.99	(0.91 to 27.46)	0.065
Month 18	15 (68.2)	14 (51.9)	1.83	(0.33 to 9.98)	0.484
Month 24	15 (68.2)	16 (59.3)	1.39	(0.26 to 7.54)	0.702
**PA cancer outcome target**					
Month 6	13 (59.1)	12 (44.4)	2.22	(0.45 to 10.92)	0.324
Month 12	8 (36.4)	9 (33.3)	1.94	(0.37 to 10.15)	0.430
Month 18	13 (59.1)	6 (22.2)	4.84	(0.93 to 25.28)	0.062
Month 24	11 (50.0)	10 (37.0)	1.71	(0.34 to 8.73)	0.515
	**Mean (SD) change in PA level**		**Estimated coefficient**	**(95% CI)**	**P**
**Change in PA level (accumulated minutes of MVPA per week)**					
Month 6	305.0 (319.5)	120.0 (114.1)	174.2	(34.7 to 313.7)	0.015
Month 12	199.3 (180.3)	93.8 (112.8)	114.1	(−32.2 to 260.5)	0.125
Month 18	291.0 (252.6)	78.5 (106.4)	179.0	(36.6 to 321.3)	0.014
Month 24	263.4 (254.1)	151.8 (174.5)	89.9	(−50.4 to 230.2)	0.206

Abbreviations: PA, physical activity; CI, confidence intervals; SD, standard deviation; MVPA, moderate-to-vigorous intensity physical activity.

^†^PA general health target: ≥30 minutes of MVPA 5 days a week.

PA cancer outcome target: ≥60 minutes of MVPA 5 days a week.

### Adverse events

At month 6, dietary interventions reduced mean daily caloric intake (−93 calories [−185-−0.5], p = 0.049), while at month 18 (Supplementary Table 3). Other than this, the dietary interventions had no significant impact on the daily caloric intake, daily protein intake and hemoglobin level at all assessment time-points. PA-associated injuries were rare in the PA intervention groups with minimal ill-effect.

## Discussion

Our theory-driven behavioral program was the first of its kind for Chinese CRC patients, which showed clear efficacy in reducing the intake of RPM over the 24-month study period without causing dietary deficiency or dietary-associated anemia. This study also demonstrated improvement in intake of refined grain by the dietary interventions, and increased PA level by the PA interventions, though the effects were not consistently significant over time. Moreover, the lack of interaction effects between dietary and PA interventions made it possible to capitalize on the statistical power gained from the factorial design assessing the intervention effects. The findings are expected to be generalizable as the CRC patients’ characteristics were similar to those reported in previous local studies^13,14^.

It is encouraging to observe a significantly higher proportion of CRC survivors achieving the dietary targets and greater reductions of RPM and RG intakes through dietary interventions. Moreover, the differences observed were clinically important and sustainable for at least 24 months. There has been an old Chinese saying that “disease enters by the mouth”. The concept of disease prevention and health improvement by dietary modification is imprinted in the Chinese culture. Our CRC survivors were more than willing to receive dietary advices after definitive cancer therapy for improving their general health status and optimizing their cancer outcomes. In line with this understanding, our dietary interventions aimed to provide knowledge on the types of food that would affect CRC outcomes and to provide practical information on how to achieve the suggested dietary targets. Personal interactions with the dietary coach through face-to-face interviews and fortnightly phone calls were welcomed by our patients as means of moral support and trouble-shooting.

This study revealed a larger reduction of RPM than RG intake. Under the staggered approach of the dietary interventions, RPM was the first target and thus the patients had more time to modify their RPM intake. Also, reducing beef and mutton is easy for CRC survivors as the former is believed to be toxic in the traditional Chinese medicine theory and the latter is not commonly eaten. Pork is the main red meat in Chinese diet. However, in Hong Kong and most part of China, there are many alternative protein sources including poultry, seafood (especially fish which is traditional Chinese diet), and a wide choice of pulses such as tofu and its derived products. In contrast, while white rice is the staple food of southern Chinese people, the whole grain alternatives such as brown and red rice are uncommon and more expensive.

Our PA interventions did not yield a significant improvement in CRC survivors’ PA levels. A possible reason was that the patients’ PA levels at baseline were already high, leaving little room for improvement. At baseline, only 49 patients had <300 minutes/week of MVPA, and the other 145 patients had between 300 and 1000 minutes/week of MVPA. These 145 patients grossly underestimated their PA levels when they self-completed the GPAQ at baseline, otherwise they could have been excluded from the study. Indeed, another study in Hong Kong that used a similar questionnaire also reported that urban older adults tended to under-report their PA levels when compared to objective accelerometer measurements^15^. Hong Kong has been objectively shown to have an extremely high “walkability score,”^16^ thus residents can accumulate substantial amounts of PA through walking^17,18^. In this regards, a recent study found that Hong Kong older adults accumulated on average 182 minutes/week of accelerometer-measured MVPA defined using cut-points developed on young Caucasians^19^. As these cut-points substantially underestimate PA in older adults, this study suggested that a large proportion of Hong Kong older adults exceed the PA recommendation of 150 minutes/week as defined by accelerometry. Excluding the physically active patients at baseline indeed substantially increased the PA effects although the effects remained statistically non-significant because of insufficient power. Future research that focuses on less physically active patients is therefore warranted.

Our patients generally complied with the assigned interventions. The interventions consisted mainly of face-to-face motivational interviews with attendance rate of over 90%, quarterly group meetings attended by 43.5% of patients, and fortnightly phone calls with successful contact rate of 72.4%. These were accomplished with persistent efforts to follow the study patients by the intervention coaches. The relatively low attendance rate of group meetings reflected our patients’ reluctance in making extra-visits to the participating hospitals, and their preference for the individual rather than the group intervention modality. One possible way to improve the attendance would be to schedule the group meetings and patients’ clinic visits in succession on the same days.

There have been studies reporting the protective effects of dietary and PA interventions on the development of CRC. However, the relationship between these behavioral change and cancer recurrence had not been well studied. The behavioral interventions evaluated in this study are complex; for which evaluation guidelines recommended the importance of assessing their feasibility^20^. This has been demonstrated in this study, and future studies can further investigate the effects of the interventions on cancer outcomes.

This study has some limitations. First, assessing patients’ eligibility by self-reported GPAQ may underestimate the actual baseline PA levels. Future studies may consider alternative approaches, e.g. the use of accelerometers or pedometers to confirm the patient’s eligibility. Second, it was difficult to gather the patients for the group meetings. Some incentives for motivating patients to participate in these meetings may be considered in future studies. Third, all the patients recruited in this trial are ethnic Chinese, hence the findings may not be directly generalizable to other ethnic groups. Future trials on other populations, especially those in the western world, are warranted.

In conclusion, the “Moving Bright, Eating Smart” program could modify CRC survivors’ dietary habits. Further work is required to assess if this intervention program could also significantly increase PA levels, especially in those with low baseline levels. Our findings are essential for the design of a larger definitive RCT in the future, which would determine the effects of behavioral interventions in reducing CRC recurrence and mortality of CRC survivors.

## Materials and Methods

This was a multi-center RCT with a 2 × 2 factorial design comparing dietary and PA interventions with usual care in adult Chinese CRC survivors. Details of the trial protocol have been published elsewhere^21^. The study protocol and informed consent forms were approved by the by the Institutional Review Board of the Hong Kong West Cluster, the Hospital Authority in Hong Kong (Reference number: UW 12-478) and site-specific approval provided by other participating centres (Island East reference number: HKEC 2012-068; and Kowloon West reference number: KW/EX-13-002 (59-02)). The RCT has been registered with ClinicalTrials.gov (NCT01708824) on 11/10/2012. Informed consent was obtained from all participants. All methods were performed in accordance with the relevant guidelines and regulations.

### Patient recruitment

We planned to recruit 224 patients with histologically confirmed CRC, aged ≥18 years and within one year of completion of main cancer treatment from the surgical and oncological departments of four public hospitals in Hong Kong. The sample size was tailored to the assessment of intervention effects on the percentage of patients reaching the corresponding dietary or PA targets. Specifically, for assessing the effect of dietary interventions on the dietary targets, we assumed that 10% of the patients not receiving the interventions would achieve their required targets. We also assumed that 25% of those who received the interventions would meet the targets, i.e. an effect size of 15%. With a power of 80% and a significance level of 5%, 200 patients were required. Using the same criteria, the assessment of PA interventions on the PA targets would need 200 patients. Assuming a 10% dropout rate, we aimed to recruit 224 patients (56 per group).

To identify eligible patients, a colorectal surgeon (JHWC) reviewed the medical records of CRC patients from the participating hospitals within the study period. Potentially eligible patients were invited to be assessed by a validated food frequency questionnaire (FFQ) and the Global Physical Activity Questionnaire (GPAQ) in their next clinic follow-up visits^22,23^. Those who had already met the dietary and/or PA targets (defined below) as reflected by the FFQ and GPAQ were excluded, while those with confirmed eligibility were asked to provide written informed consent for study participation.

Consented eligible patients were allocated to either Group A (dietary and PA interventions), Group B (dietary only), Group C (PA only), or Group D (usual care without intervention) in equal allocations using a randomization schedule generated before patient recruitment by staff not involved in the study. We used block randomization with randomly selected block-sizes between 8 and 16. Another staff not involved in the study kept the schedule and was phoned for group allocation when a patient was recruited.

### Interventions

The intervention program was based on the Theory of Planned Behavior (TPB) and the Health Action Process Approach (HAPA)^24,25^. Patients allocated to Groups A, B and C received the interventions for 12 months and were followed for another 12 months.

The interventions included individual face-to-face motivational interviews (two sessions for Groups A and B and one session for Group C), fortnightly motivational phone calls, mailed monthly stage-of-change matched educational pamphlets, mailed quarterly newsletters, and quarterly group meetings. A staggered approach was adopted to change the patients’ dietary habits: patients receiving dietary interventions were asked to gradually reduce their intake of RPM and to replace them with other protein sources. Once in the HAPA “action stage” for RPM intake for one month, the patients were asked to gradually reduce their intake of RG and replace them with whole grains. Throughout the intervention period, the patients self-monitored their changes in intakes of RPM, RG and whole grains by completing monthly diary logs. Similarly, patients receiving PA interventions were asked to progressively increase their PA levels to 60 minutes of moderate-to-vigorous intensity PA (MVPA) 5 days a week. They were provided with pedometers and monthly PA logs for tracking their changes in PA. Patients in Group A received an integrated version of both dietary and PA interventions rather than two separate interventions to avoid duplicated information and intervention overload.

Patients in the usual care group received 5 pamphlets by post at regular intervals in the first 12 months. These contained general health advice that encouraged healthy lifestyles by eating a wide variety of food, more fruit and vegetables, increasing PA levels, quitting smoking and avoiding alcohol abuse. Such information was obtained from public sources including the websites of the World Health Organization and the Department of Health in Hong Kong.

### Outcome assessment

Patients were assessed before interventions/usual care (baseline) and at every 6 months for 24 months. All outcomes were assessed by staff blinded to the patients’ group allocation.

The primary outcomes included whether the patients could achieve four behavioral targets: (i) RPM target: weekly intake of <5 servings, including <2 servings of processed meat, (ii) RG target: daily intake <2 servings, (iii) PA general health target: 30 minutes of MVPA 5 days a week, and (iv) PA cancer outcome target: 60 minutes of MVPA 5 days a week. The dietary outcomes were assessed by FFQ, while PA (MVPA) was measured by an accelerometer using standard validated protocols^19,26^. The changes in these measures from baseline to the follow-up assessment time-points were considered as secondary efficacy outcomes. Patients’ compliance and side effects from the interventions, including dietary deficiency, dietary-associated anemia and PA-associated injuries were also examined^21^.

### Statistical analysis

The primary outcomes of meeting the behavioral targets were analyzed based on the intention-to-treat principle. Patients with missing outcome measures considered not achieving the corresponding targets. To assess the effects of the dietary and PA interventions, generalized mixed effects models with logit link were used with adjustment for the corresponding baseline value, stoma status and study center. The differences in the effects of the dietary interventions (on the outcomes) between patients receiving and those not receiving PA interventions, and the differences in the effects of dietary and PA interventions over time were assessed by interaction terms. If they were not statistically significant, the overall effects of dietary and PA interventions were estimated. Otherwise, linear contrasts were used to estimate the intervention effects for different groups or time points. To deal with the potential problem due to multiple time-points comparison, we used an overall p-value to control multiplicity. That is, if the overall p-value was considered as insignificant, none of the corresponding multiple time-points comparisons would be considered as significant whether or not their p-values were considered as significant at the same nominal level of significance. Moreover, we also applied the Holm’s procedure to adjust the p-values for correcting the multiple comparisons^27^. The analysis was repeated by imputing the missing outcome measures using multiple imputation.

For the analysis of the changes in the dietary consumption and PA levels, we only included those patients with observed data. Linear mixed effects models were used to estimate the dietary and PA effects after adjusting for the baseline value, stoma status and study center. Interaction effects were assessed as in the primary analysis. Changes in caloric intake, protein intake and hemoglobin level were also evaluated for any side effects. Since some participants already met the PA cancer outcome target at baseline, i.e., had ≥300 minutes/week of accelerometer-assessed MVPA, a subgroup analysis was conducted after removing these patients.

All estimates were accompanied with 95% confidence intervals (CI), and 5% significance level was used in all statistical tests. All statistical analyses were conducted in SAS version 9.4.

## Electronic supplementary material

Supplementary Tables
