# Supplementary material for: Dietary and Physical Activity Interventions for Colorectal Cancer Survivors: A Randomized Controlled Trial
Source: Sci Rep. 2018 Apr 10;8:5731. doi: 10.1038/s41598-018-24042-6 (PMC5893594; doi:10.1038/s41598-018-24042-6)
Supplement: Supplementary file 1 — Supplementary Tables [file 41598_2018_24042_MOESM1_ESM.docx]

**Dietary and Physical Activity Interventions for Colorectal Cancer Survivors:**

**A Randomized Controlled Trial**

CF Lee^1^, Judy WC Ho^2^, Daniel YT Fong^3^, Duncan J Macfarlane^1^,

Ester Cerin^1,4^, Antoinette M Lee^5^, Sharron Leung^6^, Wynnie YY Chan^7^, Ivy PF Leung^8^,

Sharon HS Lam^2^, Natural Chu^2^, Aliki J Taylor^9^, Kar-keung Cheng^9^

**Affiliations**

1. School of Public Health, The University of Hong Kong, Hong Kong
2. Department of Surgery, The University of Hong Kong, Hong Kong
3. School of Nursing, The University of Hong Kong, Hong Kong
4. Institute for Health & Ageing, Australia Catholic University, Australia
5. Department of Psychology, The University of Hong Kong, Hong Kong
6. School of Nursing, Hong Kong Baptist Hospital, Hong Kong
7. School of Professional and Continuing Education, The University of Hong Kong, Hong Kong
8. Department of Dietetics, Queen Elizabeth Hospital, Hong Kong
9. Institute of Applied Health Research, University of Birmingham, United Kingdom

**Correspondence author**

Dr Daniel Yee Tak Fong

School of Nursing, The University of Hong Kong

4/F, William MW Mong Block, 21 Sassoon Road, Pokfulam, Hong Kong

Email: [dytfong@hku.hk](mailto:dytfong@hku.hk)

Tel: +852 3917 6645

Fax: +852 2872 6079

**Supplementary Table 1.** Number of patients meeting, not meeting and did not assess the dietary and PA targets at various time points

| Outcomes^†^ | Group A  Dietary & PA | | | Group B  Dietary only | | | Group C  PA only | | | Group D  Usual care | | |
| --- | --- | --- | --- | --- | --- | --- | --- | --- | --- | --- | --- | --- |
|  | Meeting | Not meeting | Did not assess | Meeting | Not meeting | Did not assess | Meeting | Not meeting | Did not assess | Meeting | Not meeting | Did not assess |
| Red/processed meat target |  |  |  |  |  |  |  |  |  |  |  |  |
| Baseline | 14 | 41 | 0 | 16 | 40 | 0 | 10 | 46 | 0 | 16 | 40 | 0 |
| Month 6 | 28 | 21 | 6 | 31 | 17 | 8 | 15 | 37 | 4 | 14 | 34 | 8 |
| Month 12 | 29 | 17 | 9 | 32 | 10 | 14 | 18 | 32 | 6 | 15 | 28 | 13 |
| Month 18 | 35 | 14 | 6 | 32 | 14 | 10 | 15 | 34 | 5 | 20 | 32 | 4 |
| Month 24 | 30 | 16 | 9 | 28 | 17 | 11 | 20 | 30 | 6 | 13 | 34 | 9 |
| Refined grain target |  |  |  |  |  |  |  |  |  |  |  |  |
| Baseline | 6 | 49 | 0 | 7 | 49 | 0 | 7 | 49 | 0 | 5 | 51 | 0 |
| Month 6 | 24 | 25 | 6 | 33 | 15 | 8 | 18 | 34 | 4 | 16 | 32 | 8 |
| Month 12 | 20 | 26 | 9 | 28 | 14 | 14 | 21 | 29 | 6 | 17 | 26 | 13 |
| Month 18 | 21 | 28 | 6 | 23 | 23 | 10 | 20 | 31 | 5 | 20 | 32 | 4 |
| Month 24 | 20 | 26 | 9 | 25 | 20 | 11 | 14 | 36 | 6 | 16 | 31 | 9 |
| PA general health target |  |  |  |  |  |  |  |  |  |  |  |  |
| Baseline | 47 | 4 | 4 | 46 | 3 | 7 | 46 | 3 | 7 | 50 | 4 | 2 |
| Month 6 | 40 | 0 | 15 | 33 | 3 | 20 | 41 | 1 | 14 | 39 | 0 | 17 |
| Month 12 | 34 | 2 | 19 | 25 | 2 | 29 | 35 | 0 | 21 | 28 | 2 | 26 |
| Month 18 | 38 | 1 | 16 | 29 | 3 | 24 | 37 | 1 | 18 | 34 | 1 | 21 |
| Month 24 | 34 | 1 | 20 | 27 | 2 | 27 | 37 | 0 | 19 | 37 | 0 | 19 |
| PA cancer outcome target |  |  |  |  |  |  |  |  |  |  |  |  |
| Baseline | 41 | 10 | 4 | 34 | 15 | 7 | 37 | 12 | 7 | 42 | 12 | 2 |
| Month 6 | 36 | 4 | 15 | 30 | 6 | 20 | 40 | 2 | 14 | 34 | 5 | 17 |
| Month 12 | 31 | 5 | 19 | 22 | 5 | 29 | 30 | 5 | 21 | 27 | 3 | 26 |
| Month 18 | 37 | 2 | 16 | 26 | 6 | 24 | 34 | 4 | 18 | 28 | 7 | 21 |
| Month 24 | 32 | 3 | 20 | 22 | 7 | 27 | 35 | 2 | 19 | 33 | 4 | 19 |
| Abbreviations: PA, physical activity; MVPA, moderate-to-vigorous intensity physical activity.  ^†^ Red/processed meat target: <5 servings of red and processed meat per week, including <2 servings of processed meat  Refined grain target: <2 servings of refined grain per day  PA general health target: ≥30 minutes of MVPA 5 days a week  PA cancer outcome target: ≥60 minutes of MVPA 5 days a week | | | | | | | | | | | | |

**Supplementary Table 2.** Mean and standard deviation of the dietary and PA targets at various time points

| Outcomes | Group A  Dietary & PA | Group B  Dietary only | Group C  PA only | Group D  Usual care |
| --- | --- | --- | --- | --- |
|  | Mean (SD) | Mean (SD) | Mean (SD) | Mean (SD) |
| Dietary targets |  |  |  |  |
| Red and processed meat intake (servings/week) |  |  |  |  |
| Baseline | 9.2 (6.5) | 8.3 (5.0) | 7.7 (4.3) | 8.8 (7.1) |
| Month 6 | 4.2 (3.9) | 4.4 (4.3) | 7.0 (5.2) | 7.5 (6.5) |
| Month 12 | 4.1 (4.4) | 3.3 (3.0) | 7.0 (4.9) | 7.1 (4.4) |
| Month 18 | 4.6 (4.8) | 4.3 (4.0) | 8.4 (6.5) | 7.0 (4.6) |
| Month 24 | 4.3 (3.3) | 4.8 (3.9) | 6.3 (4.4) | 7.7 (4.6) |
| Refined grain intake (servings/day) |  |  |  |  |
| Baseline | 3.0 (1.1) | 2.7 (0.9) | 2.8 (1.1) | 2.7 (0.8) |
| Month 6 | 2.0 (0.9) | 1.8 (0.6) | 2.6 (1.3) | 2.5 (1.0) |
| Month 12 | 2.2 (1.1) | 1.7 (0.6) | 2.4 (1.0) | 2.3 (0.9) |
| Month 18 | 2.2 (0.8) | 1.9 (0.7) | 2.4 (1.0) | 2.8 (0.8) |
| Month 24 | 2.1 (0.7) | 1.9 (0.6) | 2.4 (0.9) | 2.4 (0.7) |
| PA targets |  |  |  |  |
| PA level (accumulated minutes/week of MVPA) |  |  |  |  |
| Baseline | 534.2 (329.3) | 498.1 (316.8) | 460.8 (239.6) | 473.3 (267.3) |
| Month 6 | 680.5 (334.6) | 583.1 (319.7) | 641.7 (303.2) | 639.5 (333.2) |
| Month 12 | 618.6 (268.2) | 535.9 (268.5) | 570.1 (204.2) | 616.6 (310.0) |
| Month 18 | 730.6 (331.4) | 608.4 (411.4) | 631.8 (281.4) | 676.2 (367.4) |
| Month 24 | 730.9 (382.6) | 576.2 (354.4) | 680.5 (259.8) | 642.4 (294.7) |
| Abbreviations: SD, standard deviation; PA, physical activity; MVPA, moderate-to-vigorous intensity physical activity. | | | | |

**Supplementary Table 3.** Potential adverse events due to the dietary interventions

|  | Mean (SD) | | Difference | (95% CI) | P |
| --- | --- | --- | --- | --- | --- |
|  | Groups A+B | Groups C+D | Dietary interventions |  |  |
| Daily caloric intake (Kcal) |  |  |  |  |  |
| Baseline | 1524 (382) | 1534 (452) |  |  |  |
| Month 6 | 1366 (386) | 1472 (403) | -93 | (-185 to -0.5) | 0.049 |
| Month 12 | 1504 (351) | 1520 (356) | -34 | (-129 to 62) | 0.489 |
| Month 18 | 1531 (448) | 1566 (400) | -22 | (-115 to 70) | 0.633 |
| Month 24 | 1534 (378) | 1531 (385) | 17 | (-77 to 111) | 0.726 |
| Daily protein intake (g) |  |  |  |  |  |
| Baseline | 72.0 (20.5) | 75.1 (27.4) |  |  |  |
| Month 6 | 69.3 (17.7) | 71.2 (22.9) | -0.02 | (-5.07 to 5.03) | 0.994 |
| Month 12 | 70.3 (20.8) | 71.3 (21.0) | 0.25 | (-4.95 to 5.45) | 0.926 |
| Month 18 | 68.6 (20.3) | 72.1 (22.3) | -1.29 | (-6.34 to 3.75) | 0.615 |
| Month 24 | 71.5 (17.6) | 69.4 (21.5) | 4.31 | (-0.83 to 9.45) | 0.100 |
| Hemoglobin level (g/dL) |  |  |  |  |  |
| Baseline | 2.8 (1.0) | 2.7 (0.9) |  |  |  |
| Month 6 | 1.9 (0.8) | 2.5 (1.1) | -0.04 | (-0.39 to 0.31) | 0.821 |
| Month 12 | 2.0 (0.9) | 2.4 (1.0) | -0.11 | (-0.47 to 0.24) | 0.527 |
| Month 18 | 2.1 (0.8) | 2.3 (0.9) | -0.13 | (-0.47 to 0.21) | 0.458 |
| Month 24 | 2.0 (0.7) | 2.4 (0.8) | 0.08 | (-0.27 to 0.42) | 0.670 |

**Supplement Figure 1.** Effects of dietary interventions on achieving (a) Red/processed meat target and (b) Refined grain target, and effects of physical activity interventions on achieving (c) PA general health target and (d) PA cancer outcome target

(a)


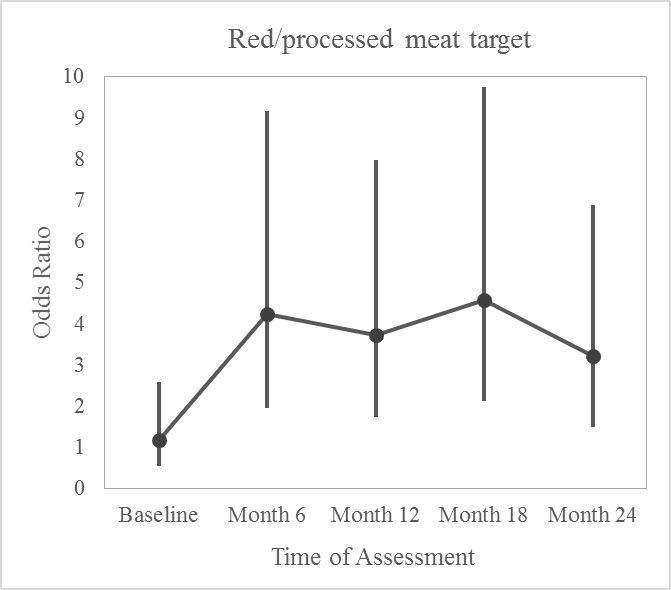


(b)

**Supplement Figure 1.** Effects of dietary interventions on achieving (a) Red/processed meat target and (b) Refined grain target, and effects of physical activity interventions on achieving (c) PA general health target and (d) PA cancer outcome target (continued)

(c)

(d)

**Supplementary Figure 2.** Effects of dietary interventions on mean changes in (a) Red and process meat intake and (b) Refined grain intake, and effects of physical activity interventions on mean change in (c) MVPA

(a)

(b)

**Supplementary Figure 2.** Effects of dietary interventions on mean changes in (a) Red and process meat intake and (b) Refined grain intake, and effects of physical activity interventions on mean change in (c) MVPA (continued)

(c)
